# Heat Shock Protein 70 Constitutes a Promising Novel Biomarker in Differential Diagnosis between Takotsubo Syndrome and Non-ST-Segment Elevation Myocardial Infarction

**DOI:** 10.3390/jcm13144152

**Published:** 2024-07-16

**Authors:** Ozan Demirel, Vera Paar, Philipp Tolnai, Albert Topf, Uta C. Hoppe, Michael Lichtenauer, Moritz Mirna

**Affiliations:** 1 Clinic of Internal Medicine II, Department of Cardiology, Paracelsus Medical University of Salzburg, 5020 Salzburg, Austriav.paar@salk.at (V.P.);; 2 Hospital Villach, Department of Internal Medicine, 9500 Villach, Austria

**Keywords:** biomarker, HSP70, acute coronary syndrome, ACS, NSTEMI, Takotsubo cardiomyopathy, TTS

## Abstract

(1) **Background**: Due to similar clinical presentation and a lack of specific biomarkers, initial differentiation between Takotsubo syndrome (TTS) and non-ST-segment elevation myocardial infarction (NSTEMI) remains challenging in daily practice. Heat Shock Protein 70 (HSP70) is a novel biomarker that is recognized for its potential in the diagnosis and differentiation of cardiovascular conditions. (2) **Methods**: Data from a total of 156 patients were analyzed (32.1% NSTEMI, 32.7% TTS, and 35.3% controls). Serum concentrations of HSP70 were determined using ELISA and compared between patients and controls. ROC curve analysis, logistic regression analysis and propensity-score-weighted logistic regression were conducted. (3) **Results**: Concentrations of HSP70 were highest in patients with TTS (median 1727 pg/mL vs. ACS: median 1545 pg/mL vs. controls: median 583 pg/mL, *p* < 0.0001). HSP70 was predictive for TTS in binary logistic regression analysis (B(SE) = 0.634(0.22), *p* = 0.004), which even remained significant after correction for possible confounders in propensity-score-weighted analysis. ROC curve analysis also revealed a significant association of HSP70 with TTS (AUC: 0.633, *p* = 0.008). (4) **Conclusions**: Based on our findings, HSP70 constitutes a promising biomarker for discrimination between TTS and NSTEMI, especially in combination with established cardiovascular biomarkers like pBNP or high-sensitivity cardiac troponin.

## 1. Introduction

Cardiovascular diseases remain the leading cause of morbidity and mortality worldwide [1]. Early diagnosis and differentiation of these disease entities are crucial, not only to initiate appropriate therapeutic interventions but also to reduce the associated high mortality rates. The overall incidence of Takotsubo syndrome (TTS) among patients presenting with suspected acute coronary syndrome (ACS) is estimated to be between 1% and 2%, while the majority of TTS cases are constituted by postmenopausal women [2,3,4]. Because of their similar clinical presentation, initial differential diagnosis between TTS and non-ST-segment elevation myocardial infarction (NSTEMI) remains a major challenge in clinical practice.

Among the various parameters studied to understand and diagnose cardiovascular diseases, serum biomarkers have emerged as invaluable diagnostic tools, for example in heart failure (HF) or ACS [5,6]. An emerging biomarker in cardiovascular medicine is Heat Shock Protein 70 (HSP70), which constitutes a stress-inducible molecular chaperone involved in a variety of cellular functions, including protein folding, damage repair, and remodeling processes [7]. Because of its induction under stress conditions [8], HSP70 appears to be a suitable biomarker in the differential diagnosis of ACS and TTS since the latter has a known pathophysiological association with catecholamine-induced myocardial injury [9]. Furthermore, HSP70 promotes the maintenance of protein homeostasis and enhances cell survival following a multitude of cellular stressors [10]. HSP70 is also associated with other stress-related conditions. A reduction in HSP70 expression with increasing age seems to compromise the stress response, rendering them more susceptible to stress-related damage and disease [11]. HSP70 also inhibits multiple apoptosis pathways, which might provide survival advantages to tumor cells [12].

In humans, HSP70 levels have been postulated to correlate with the presence, early diagnosis, and disease severity of HF [13,14,15]. Moreover, HSP70 even has potential as a diagnostic and therapeutic target in the context of cardiovascular and cerebrovascular disorders [16,17]. In particular, HSP70 has been shown to play a role in the development of atherosclerosis. While it shows protective effects against cellular stress and may reduce the risk of coronary artery disease, its involvement in immune responses and potential degradation in plaques add complexity to its role [18]. Furthermore, the balance between extracellular and intracellular HSP70—quantified as the eHSP70/iHSP70 ratio—has the potential to act as a biomarker of inflammatory status and endothelial dysfunction in atherosclerosis [19]. In addition, HSP70 could potentially protect cells against oxidative stress induced by modified low-density lipoprotein (LDL) particles [20]. Moreover, hypertension is linked to inflammation and stress-induced HSP70 production—with anti-HSP70 antibodies suggesting an autoimmune component—and experimental models have demonstrated that an immune response to HSP70 can cause hypertension, whereas tolerance to HSP70 can prevent it [21,22]. Although there is no significant difference in HSP70 levels between individuals with atrial fibrillation (AF) and those without, increased expression of this protein in atrial tissue is associated with a reduced likelihood of developing postoperative AF, indicating a protective function [23].

Given the diverse functions of HSP70 in cardiac tissue, it is important to understand its behavior in cardiovascular diseases and its potential benefit in the differential diagnosis of clinically similar disease entities, such as TTS and NSTEMI. Therefore, this study aims to investigate the serum concentrations of HSP70 in patients diagnosed with TTS and NSTEMI versus controls. We seek to assess the predictive power of HSP70 in differentiating between these cardiovascular conditions. The outcomes of this study may provide valuable insights into the potential utility of HSP70 as a diagnostic biomarker in cardiovascular medicine.

## 2. Materials and Methods

This study constitutes a retrospective analysis of prospectively collected data, which were acquired between 2019 and 2024 at the University Hospital of Salzburg, Austria.

### 2.1. Patients

Patients were enrolled according to the following inclusion criteria: (1) diagnosis of TTS with fulfilment of the respective InterTAK Diagnostic Criteria [24]; (2) NSTEMI, according to the current guidelines of the European Society of Cardiology [6]; or patients with chest pain who presented to the emergency department of our hospital and in whom acute myocardial infarction (AMI) was excluded by serial troponin measurements. The latter constituted the control group.

Inclusion criteria required patients to be aged 18 years or older. Patients with ST-segment elevation myocardial infarction (STEMI) or those classified as very high-risk NSTE-ACS were excluded. Additionally, patients unable to provide written informed consent, who were incapacitated, or under the age of 18 were also excluded.

Blood samples were collected from a cubital vein under controlled venous stasis within 24 h of admission to the hospital. The tubes were centrifuged for 20 min at 2000× *g* after clotting of the serum samples and the supernatant was frozen at −80 °C until further analysis. Additionally, routine blood analysis was performed at the time of initial study sample collection according to the local clinical standards.

### 2.2. Measurement of Serum HSP70 Concentrations

Serum HSP70 analysis was conducted using a commercially available enzyme-linked immunosorbent assay (ELISA) kit for HSP70/HSPA1A (Human HSP70/HSPA1A DuoSet ELISA, DY1663-05, R&D Systems, Minneapolis, MN, USA). The assay was performed according to the manufacturer’s instructions. In brief, 96-well plates (Thermo Fisher, Waltham, MA, USA) were incubated with a specific capture antibody, which was incubated overnight at room temperature. The next day, the plates were washed three times with 1x phosphate-buffered saline (PBS), with 0.05% Tween 20 (Carl Roth, Karlsruhe, Germany), and blocked with 1x PBS supplemented with 1% bovine serum albumin (BSA, Carl Roth, Germany). After a second washing step, the standard was applied to the plate in a defined serial dilution, and 100 µL of the different serums were pipetted into the wells. After an incubation of 2 h, plates were washed again, and the provided biotin-labelled detection antibody was applied. After further two hours of incubation and a subsequent washing step, a streptavidin–horseradish–peroxidase (HRP) solution (provided within the kit) was added and incubated for 20 min. Finally, the plates were washed one last time before the substrate tetramethylbenzidine (TMB; Sigma Aldrich, Burlington, MA, USA) was added to achieve a colour reaction. The reaction was stopped by adding 50 µL of 2 N sulphuric acid solution (H_2_SO_4_, Sigma Aldrich, USA) and the optical density (OD) was measured at 450 nm using an ELISA plate reader (iMark Microplate Absorbance Reader, Bio-Rad Laboratories, Wien, Austria). Protein concentrations were automatically calculated by Microplate Manager 6 (MPM6, version 6.3) software (Bio-Rad Laboratories, Wien, Austria) using the OD of the standard curve.

### 2.3. Statistical Analysis

Statistical analysis was conducted with R (version 4.2.1., R Core Team (2013), R Foundation for Statistical Computing, Vienna, Austria; http://www.R-project.org/ (accessed on 1 May 2024) using the packages ‘Rcmdr’, ‘ggplot2’, ‘pastecs’, ‘Hmisc’, ‘ggm’, ‘polycor’, ‘QuantPsyc’, ‘glmnet’, ‘Matching’, ‘MatchIt’, ‘optmatch’, ‘RItools’, ‘Rcpp’, ‘twang’, ‘survey’, and ‘stddidff’; SPSS (Version 23.0, IBM, Armonk, New York, NY, USA); and GraphPad Prism (version 5.01, GraphPad Software, La Jolla, CA, USA). Data distribution was assessed by Kolmogorov–Smirnov test, whereas kurtosis and skew were assessed visually. As data were not normally distributed, median ± interquartile range (IQR) are depicted in the manuscript. Medians were compared by Kruskal–Wallis test with Dunn’s post hoc test. Categorical data were analyzed by Fisher’s exact test. Receiver operating characteristic (ROC) analysis was conducted and a cut-off for the presence of TTS was determined by calculating the Youden index [25]. To account for possible covariate imbalances, propensity score weighting of the groups was conducted using Generalized Boosted Models (GBM) and the Average Treatment Effect (ATE) [26]. Prior to GBM, continuous data were transformed to z-scores to assure standardization. Covariates with statistically significant differences between groups were then included in propensity score weighing (age, sex, hypertension, BMI (body mass index), creatinine, CRP (C-reactive protein), pBNP (pro-brain natriuretic peptide), high-sensitive troponin T (hsTnT), and EF (ejection fraction)). After balancing, weighted binary logistic regression analysis was performed using the ‘survey’ package of R. A *p*-value of <0.05 was considered statistically significant.

## 3. Results

In total, 156 patients were enrolled in the present study. Of these, 32.7% (n = 51) had TTS, 32.1% (n = 50) had NSTEMI, and 35.3% (n = 55) were controls.

Baseline characteristics are depicted in Table 1. Patients with TTS were significantly older (TTS: median 69 years vs. NSTEMI: median 67 years vs. controls: median 59 years, *p* = 0.001) and more often female (TTS: 94.1% vs. NSTEMI: 34.0% vs. controls: 45.5%, *p* < 0.0001). In contrast, arterial hypertension was more prevalent in patients with NSTEMI (86.0% vs. TTS: 74.5% vs. controls: 54.5%, *p* = 0.001) and body mass index (BMI) was also higher in these patients (median 27.8 kg/m^2^ vs. TTS: median 25.1 kg/m^2^ vs. controls: 26.2 kg/m^2^, *p* = 0.018). HSP70 concentrations showed a weak correlation with CRP (Rs = 0.172, *p* = 0.035) and hsTnT (Rs = 0.191, *p* = 0.019) in the total cohort.

### 3.1. Serum Concentrations of HSP70

**Figure 1:**
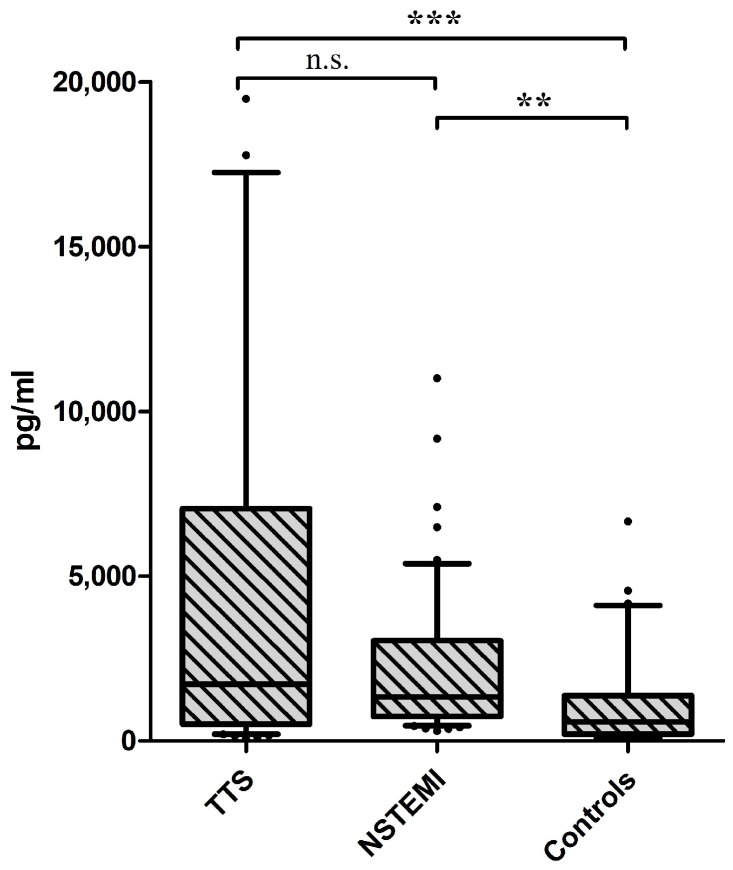
Boxplots of serum concentrations of Heat Shock Protein 70 (HSP70) in the three investigated groups. ** indicates a *p* of <0.01 and *** indicates a *p* of <0.001, n.s. = not significant.

Serum concentrations of HSP70 were significantly different between the three groups investigated, with the highest levels observed in patients with TTS and the lowest in controls (TTS: median 1727 pg/mL (IQR 508–7049) vs. NSTEMI: median 1341 pg/mL (IQR 736–3040) vs. controls: median 583 pg/mL (IQR 210–1378), *p* < 0.0001, see Figure 1). Of note, there was a statistically significant difference between patients with NSTEMI and controls (*p* = 0.001), as well as TTS and controls (*p* < 0.0001), but not between NSTEMI and TTS (*p* = 0.485; see Figure 1).

### 3.2. ROC Curve Analysis

**Figure 2:**
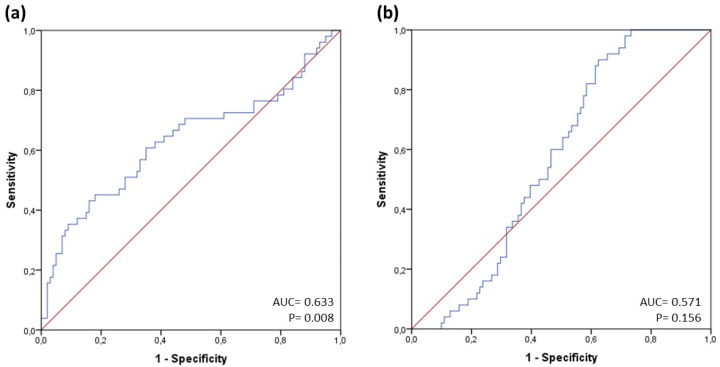
ROC curves of levels of Heat Shock Protein 70 (HSP70) for presence of (**a**) Takotsubo syndrome (TTS) and (**b**) non-ST-segment elevation myocardial infarction (NSTEMI). Abbreviations: AUC = area under the curve; ROC = receiver operating characteristic.

ROC curve analysis was performed and the area under the curve (AUC) was calculated for the discrimination of NSTEMI and TTS in the total study cohort. HSP70 showed adequate discriminatory ability for TTS (AUC: 0.633, (95% CI 0.531–0.734), *p* = 0.008), yet no relevant association with NSTEMI in the total cohort (AUC: 0.571, (95% CI 0.482–0.660), *p* = 0.156; see Figure 2a,b).

A cut-off for discrimination of TTS was determined at ≥3276 pg/mL (specificity: 84.0%, sensitivity: 43.1%, positive predictive value (PPV): 71.0%, negative predictive value (NPV): 58.6%).

### 3.3. Binary Logistic Regression Analysis

In the univariate binary logistic regression analysis, serum concentrations of HSP70 were significantly associated with the presence of TTS but not with NSTEMI (see Table 2). Of note, the predictive ability of HSP70 for TTS was lower than that of pBNP (HSP70: B(SE) = 0.634(0.22), *p* = 0.004; pBNP: (B(SE) = 2.22(0.57), *p* < 0.0001; see Table 2).

### 3.4. Propensity-Score-Weighted Logistic Regression Analysis

In order to limit the possible influence of baseline covariate imbalances on our outcome, we additionally conducted propensity-score-weighted logistic regression analysis for sex, hypertension, age (z-score), BMI (z-score), creatinine (z-score), CRP (z-score), pBNP (z-score), and EF (z-score). Here, high levels of HSP70 were significantly associated with the prevalence of TTS (B(SE) = 0.14(0.03), *p* < 0.0001), whereas lower levels were associated with the presence of NSTEMI (B(SE) = −0.12(0.03), *p* = 0.0002).

## 4. Discussion

In clinical practice, initial differentiation between TTS and NSTEMI remains challenging because of the similar presentation of both diseases. To date, no established biomarker has been validated to safely aid in the discrimination of these two conditions. Therefore, this study aimed to assess the potential value of HSP70 in the differential diagnosis between NSTEMI and TTS.

The results of our study show that TTS patients had the highest levels of HSP70 compared to both NSTEMI patients and healthy individuals. This suggests that HSP70 may be a promising biomarker to differentiate between these conditions. Intriguingly, however, it became apparent that a combination of HSP70 and pBNP was associated with the presence of TTS, whereas hsTnT and low pBNP values were associated with NSTEMI. This observation is similar to the existing literature on BNP levels, which are also significantly higher in TTS patients compared to troponin levels. Thus, the BNP/troponin ratio has already demonstrated promise as a diagnostic tool for differentiating TTS from AMI [27]. Moreover, previous research indicated that although traditional markers of cardiac injury such as troponins are elevated in TTS, their levels are significantly lower compared to AMI [28]. Of note, the predictive ability of HSP70 for TTS was less pronounced than that of pBNP. Thus, HSP70 could add incremental diagnostic value in the differential diagnosis of these two diseases, especially when used in combination with established cardiovascular biomarkers like pBNP or hsTnT.

Despite its promise, the sensitivity and specificity of HSP70 at the identified cut-off (≥3276 pg/mL) indicate that it may be more effective in ruling in TTS rather than ruling it out. The relatively high specificity (84.0%) suggests that elevated HSP70 levels strongly indicate TTS but the moderate sensitivity (43.1%) indicates that not all TTS patients will have elevated HSP70. Therefore, HSP70 should be used in conjunction with other clinical findings and biomarkers to improve diagnostic accuracy. In the clinical setting, this could be implemented through decision algorithms, such as decision trees or risk scores, that integrate HSP70 measurements with other biomarkers such as pBNP and hsTnT. These algorithms could assist clinicians in evaluating the likelihood of TTS versus other conditions, thereby enhancing patient outcomes. Moreover, the use of point-of-care testing devices capable of simultaneously measuring multiple biomarkers could expedite the diagnostic process, accelerating accurate decision-making.

HSP70 appears to be a promising cardiac biomarker for TTS because it plays a critical role in reducing stress-induced damage to myocardial tissue, which is central to TTS pathophysiology. TTS is associated with acute emotional or physiological stress, which triggers a surge in catecholamines, such as adrenaline and noradrenaline, causing oxidative stress, calcium overload, and activation of apoptotic pathways in myocardial cells [29,30,31,32,33]. The findings of this study are consistent with previous research indicating that HSP70 is upregulated in conditions of cellular stress and myocardial injury [34,35,36,37,38]. Since HSP70 is specifically induced by stress and myocardial injury, it may provide a more targeted approach to identifying TTS, particularly in patients presenting with acute stress-related cardiac symptoms.

Intriguingly, HSP70 showed a weak correlation with CRP and hsTnT in the total study cohort. Previous studies have also shown that HSP70 levels are associated with serum uric acid and oxidative stress [39]. It is noteworthy that both TTS and preeclampsia are female diseases that share increased HSP70 levels and genetic polymorphisms [40]. Also, the risk of developing preeclampsia is associated with the uric acid to creatinine ratio [41], which might be used to aid in the diagnosis of TTS in the future.

Our study opens new avenues for future research on HSP70 as a diagnostic biomarker in cardiovascular disease. Further studies are needed to validate our findings in larger, multi-center cohorts to validate the findings across diverse populations and clinical settings. Integrating HSP70 measurements with other emerging biomarkers and advanced imaging techniques could improve diagnostic accuracy and patient outcomes in cardiovascular disease management.

## 5. Conclusions

This study highlights the potential of HSP70 as a diagnostic biomarker, particularly in the identification of TTS. While HSP70 levels are significantly elevated in TTS patients, its diagnostic accuracy is improved when used in conjunction with other established biomarkers. Further prospective validation studies are needed to establish its clinical applicability and generalizability.

## 6. Limitations

The current study constitutes a retrospective analysis of previously collected data. A prospective study approach would have been superior regarding its generated level of evidence. Moreover, we found several differences in baseline covariates between the three investigated groups. Although we performed propensity-score-weighted regression analysis to minimize an influence thereof, a possible bias cannot certainly be excluded. Additionally, the reliance on a single institution’s patient population may introduce selection bias, highlighting the need for multi-center studies to confirm these findings. The uneven group distribution may have introduced bias, suggesting that larger, more balanced cohorts are required in future research for enhanced reliability. The reader should consider these limitations when the findings of our study are interpreted.

## Figures and Tables

**Table 1:** Baseline characteristics of patients investigated. Abbreviations: BMI = body mass index, CRP = C-reactive protein, CI = confidence interval, EF = ejection fraction, hsTnT = high-sensitive troponin T, IQR = interquartile range, NSTEMI = non-ST-segment elevation myocardial infarction, pBNP = pro-brain natriuretic peptide.

	Takotsubo Syndrome			NSTEMI			Controls			
	Median	IQR	95% CI	Median	IQR	95% CI	Median	IQR	95% CI	*p*-Value
Age (years)	69	62–78	67–73	67	57–77	63–70	59	40–74	51–61	0.001
BMI (kg/m^2^)	25.1	21.8–29.2	24.1–26.9	27.8	25.6–30.4	27.1–29.8	26.2	23.5–28.5	25.4–27.8	0.018
Creatinine (mg/dL)	0.76	0.68–0.90	0.75–0.89	0.78	0.67–1.00	0.81–1.01	0.96	0.86–1.09	0.13–3.55	<0.0001
CRP (mg/dL)	0.50	0.20–0.90	0.50–2.38	3.50	0.00–7.45	3.16–9.87	0.10	0.10–0.30	0.17–0.63	<0.0001
pBNP (ng/L)	2866	665–4920	2623–4810	293	152–1142	375–1380	66	164–1466	95–1061	<0.0001
hsTnT (ng/L)	162	53–395	207–392	678	131–1223	528–2373	6	5–14	8–12	<0.0001
EF (%)	40	35–46	40–44	50	40–63	47–55	50	45–57	45–55	0.001
	**%**	**n**		**%**	**n**		**%**	**N**		***p*** **-value**
Atrial fibrillation	9.8	5		13.3	6		1.9	1		0.097
Sex (female)	94.1	48		34.0	17		45.5	25		<0.0001
Diabetes	5.9	3		23.3	10		14.5	8		0.056
Hypertension	74.5	38		86.0	43		54.5	30		0.001
Smoker	29.4	15		44.2	19		29.1	16		0.220

**Table 2:** Binary logistic regression analysis for presence of Takotsubo syndrome or NSTEMI. Abbreviations: B = regression coefficient, HSP70 = Heat Shock Protein 70, NSTEMI = non-ST-segment elevation myocardial infarction, pBNP = pro-brain natriuretic peptide, SE = standard error.

	**NSTEMI**		
	**B**	**SE**	***p*-value**
HSP70, z-score	−0.354	0.24	0.142
pBNP, z-score	−1.579	0.54	0.003
hsTnT, z-score	5.477	1.07	<0.0001
	**Takotsubo Syndrome**		
	**B**	**SE**	***p*-value**
HSP70 (pg/mL)	0.634	0.22	0.004
pBNP (pg/mL)	2.216	0.57	<0.0001
hsTnT, z-score	−0.564	0.51	0.266

## Data Availability

The data underlying this research will be shared upon reasonable request to the corresponding author.
